# Jiannao Pills alleviate depression-like behavior in chronic unpredictable mild stress-induced mice through NF-κB/NLRP3 pathway

**DOI:** 10.3389/fphar.2025.1606445

**Published:** 2025-06-19

**Authors:** Jia Pan, Jie Liu, Qingying Fan, Shiman Gao, Yuanyuan Liang, Lihan Zhang

**Affiliations:** ^1^ Institute of Pharmacology and Toxicology, Sichuan Academy of Chinese Medicine Sciences, Chengdu, China; ^2^ Department of Pharmacy, Shandong Qingdao Huangdao District People’s Hospital, Qingdao, China; ^3^ Department of Clinical Pharmacy, Women and Children’s Hospital of Qingdao University, Qingdao, China; ^4^ Institute of Clinical Basis and Literature Information, Sichuan Academy of Chinese Medicine Sciences, Chengdu, China; ^5^ School of Clinical Medicine, Chengdu University of Traditional Chinese Medicine, Chengdu, China

**Keywords:** depression, Jiannao Pills, chronic unpredictable mild stress, NF-κB, NLRP3

## Abstract

**Objective:**

Depression is a significant mental disorder that damages human health. Jiannao Pills (JNW) as a commercial mediation has demonstrated its effectiveness in depression treatment but the mechanism remains elusive. This research endeavored to examine the effects of JNW on the depression-like behavior in mice subjected to chronic unpredictable mild stress (CUMS) and to elucidate the underlying molecular mechanism.

**Methods:**

The CUMS-induced mice were utilized to evaluate the antidepressant efficacy of JNW. JNW was intragastric administered at the dosage of 0.375, 0.75 and 1.5 g/kg for 32 consecutive days. The following tests were employed to assess depression-like behavior: sucrose preference test (SPT), coat state assessment (CSA), tail suspension test (TST), forced swimming test (FST), and open field test (OFT). The concentrations of Hypothalamic-pituitary-adrenal (HPA) axis hormone, monoamine neurotransmitter and pro-inflammation cytokine was quantified by ELISA. The transcriptional levels of pro-inflammation cytokine, Bcl-2, Bax, NLRP3 and Caspase-1 was assessed by qRT-PCR. The protein expression level of Bcl-2, Bax, NF-κB p65, IκBα, p-IκBα, NLRP3 and Caspase-1 was determined by Western blot.

**Results:**

JNW could significantly enhance the sucrose preference, decrease CSA score, reduce immobility time of TST and FST and improve various aspects of behavior in OFT, including an increase in the number of entries to the central zone, the duration spent in the central zone, and the total movement distance. JNW could also suppress IL-1β, IL-6, and TNF-α production and mRNA expression, and lower the level of CRH in the hypothalamus and ACTH and CORT in the serum, increase the level of monoamine neurotransmitter in both serum and hippocampus, upregulate the mRNA and protein expression of Bcl-2 while downregulate the mRNA and protein expression of Bax. Furthermore, JNW notably suppressed NLRP3 and Caspase-1 mRNA and protein, inhibited the expression of NF-κB p65, and increased phosphorylation and degradation of IκBα.

**Conclusion:**

JNW could alleviate depression-like behavior in mice subjected to CUMS, and these effects may be mediate through NF-κB/NLRP3 pathway.

## 1 Introduction

Depression is a significant mental disorder that damages human health. It is the leading cause of “years lost” to disability, surpassing all other conditions (Smith, 2014). According to the estimation by World Health Organization, approximately 350 million people worldwide suffer from depression. In 2020, past 12-month depression was prevalent among 9.2% of individuals aged 12 and older in US (Goodwin et al., 2022), while prevalence was approximately 4% in China (Lu et al., 2021). The cost of illness associated depression is more than twice that of the comparator group (König et al., 2019). The incremental economic burden of major depression in US alone is estimated to exceed $382 billion (Greenberg et al., 2023). Hence, the management of depression holds immense social significance and economic value.

Depression is frequently characterized as a stress-related disorder, and it is widely recognized that stress arising from negative life events is crucial for its development, manifestation, and neuro-progression (Cruz-Pereira et al., 2020). Several hypotheses are proposed to elucidate the development of depression. Monoaminergic hypothesis posits that the etiological cause of depression is an impairment in the monoamine neurotransmitter system. This theory has led to the discovery of a range of drugs that now constitute the cornerstone of depression treatment (Perez-Caballero et al., 2019). Due to its crucial involvement in the stress response, it has been hypothesized that the hypothalamic-pituitary-adrenal (HPA) axis plays a major role in the onset of depression. A malfunctioning HPA axis negative feedback loop leads to a prolonged elevation of glucocorticoids, subsequently exacerbating the manifestation of depressive symptoms (Menke, 2024). However, these hypotheses are inadequate to fully elucidate the pathological mechanisms underlying depression, as well as the pharmacological mechanisms of antidepressants.

Over the past few years, the significance of inflammation in both the initiation and progression of depression has received growing attention. Recent research has revealed a crucial role for pro-inflammatory cytokines, including interleukin (IL)-1β, IL-6, and tumor necrosis factor (TNF)-α, in stress-induced depression. These cytokines have been found to contribute to the development of depression by influencing neuroinflammation, monoamine neurotransmitters, the HPA axis, and neuroplasticity (Harsanyi et al., 2022). Clinical and preclinical studies indicate that the nucleotide-binding oligomerization domain (NOD)-like receptor protein 3 (NLRP3) inflammasome serves as a molecular mechanism through which psychological stress stimuli are translated into inflammatory responses (Kouba et al., 2022). The NLRP3 inflammasome is a prominent inflammatory vesicle, comprising a sensor known as NLRP3, an adaptor protein called apoptosis-associated speck-like protein containing a CARD (ASC), and an effector enzyme, Caspase-1. Caspase-1 is critical in regulating the processing of inflammatory factor precursors, such as pro-IL-1β and pro-IL-18, converting them into their active forms, IL-1β and IL-18 (Ma, 2023). These findings suggest that NLRP3- mediated inflammation exerts a significant influence on the pathogenesis of depression.

Traditional Chinese Medicine (TCM) has exhibited notable clinical efficacy and impressive safety in the treatment of depression (Dang et al., 2024). Moreover, it can achieve anti-depressant effects via diverse mechanisms (Zhuang et al., 2023), indicating immense potential for the development of innovative anti-depressant medications. Jiannao Pills (JNW) is a commercially available medication for nourishing the brain, enhancing mental acuity, calming the heart and soothing the spirit, which is consist of *Angelicae sinensis radix*, *Bambusae concretio silicea*, *Cistanches herba*, and 16 other medicinal materials. Given its alignment with the etiological and pathological understandings of depression outlined in Traditional Chinese Medicine (TCM) theory, JNW has been employed in the treatment of depression and has demonstrated its efficacy. However, the specific mechanism responsible for JNW’s effectiveness remains elusive.

Hence, the current research aimed to assess the impact of JNW on the depression-like behavior in chronic unpredictable mild stress-induced mice. Additionally, it intended to evaluate the effect of JNW on pro-inflammation cytokine production, the HPA axis hormone, monoamine neurotransmitter and hippocampal apoptosis, and further to investigate its mechanism with a focus on NF-κB/NLRP3 pathway.

## 2 Materials and methods

### 2.1 Preparation of JNW

JNW (batch number: 1721012, date of production: 2021.11) were manufactured by SPH Qingdao Growful Pharmaceutical Co., Ltd., in. According to the Chinese Pharmacopoeia, JNW is prepared as follows: *Angelicae sinensis radix* (25 g), *Bambusae concretio silicea* (10 g), *Cistanches herba* (20 g), *Dens draconis* (10 g), *Dioscoreae rhizome* (20 g), *Succinum* (10 g), *Schisandrae chinensis fructus* (15 g), *Gastrodiae rhizome* (5 g), *Platycladi semen* (4 g), *Salviae miltiorrhizae radix et rhizome* (5 g), *Alpiniae oxyphyllae fructus* (15 g), *Ginseng radix et rhizome* (5 g), *Polygalae radix* (10 g), *Chrysanthemi flos* (5 g), *Anemones altaicae rhizome* (10 g), *Haematitum* (3.75 g)*, Arisaema cum bile* (10 g), *Ziziphi spinosae semen* (40 g) and *Lycii fructus* (20 g) are powdered and sterilized, and then mixed with sodium alginate (12 g) to form pills, which are subsequently coated with *Haematitum* (3.75 g) and *Amygdali resina* (1 g). The final yield is about 1583 pills (0.15 g per pill). All drugs were validated taxonomically according the pharmacopoeia and acquired in full compliance with legal regulations. The finished products were tested according to the pharmacopoeia and complied with the specified requirements. HPLC analysis revealed that this batch of JNW contained 0.23 mg of Schisandrol A per 5 pills, which meets the pharmacopoeial standards (not less than 0.16 mg per 5 pills). The chemical composition detected by UHPLC/QE/MS was reported previously (Pan et al., 2024). The pills were dispersed in distilled water and diluted to the required concentration. The preparation is made fresh daily prior to administration.

### 2.2 Animal

Eight-week-old male Balb/c mice, weighting 18–22 g, were acquired from SPF (Beijing) Biotechnology Co., Ltd. Prior to the experiments, all mice were housed in a stable environment maintained at a temperature of 23°C ± 2°C, humidity 50% ± 10%, and a 12 h dark/light cycle, with unlimited access to water and standard food. The animal experiments were approved by the Committee of Animal Care of Sichuan Academy of Chinese Medicine Sciences, under the permit number SYLL(2023)-030.

### 2.3 Reagents

Fluoxetine capsule were purchased from Shanxi C&Y Pharmaceutical Group Co., Ltd. Mouse serotonin (5-HT), norepinephrine (NE), corticotropin releasing hormone (CRH), adrenocorticotropic hormone (ACTH), Corticosterone (CORT), IL-1β, IL-6, TNF-α ELISA kit was obtained from Jiangsu Meimian Industrial Co., Ltd. Total RNA extraction kit and the first cDNA synthesis kit was obtained from TransGen Biotech (Beijing, China). SYBR green qPCR mix was obtained from CWBio (Jiangsu, China). The antibodies targeting NLRP3, Caspase-1, NF-κB p65, IκBα, p-IκBα, Bax, Bcl-2 and β-actin, along with the HRP-conjugated secondary antibodies, were all procured from Proteintech (Wuhan, China). ECL reagent was obtained from Solarbio (Beijing, China).

### 2.4 Groups and drug administration

**FIGURE 1:**
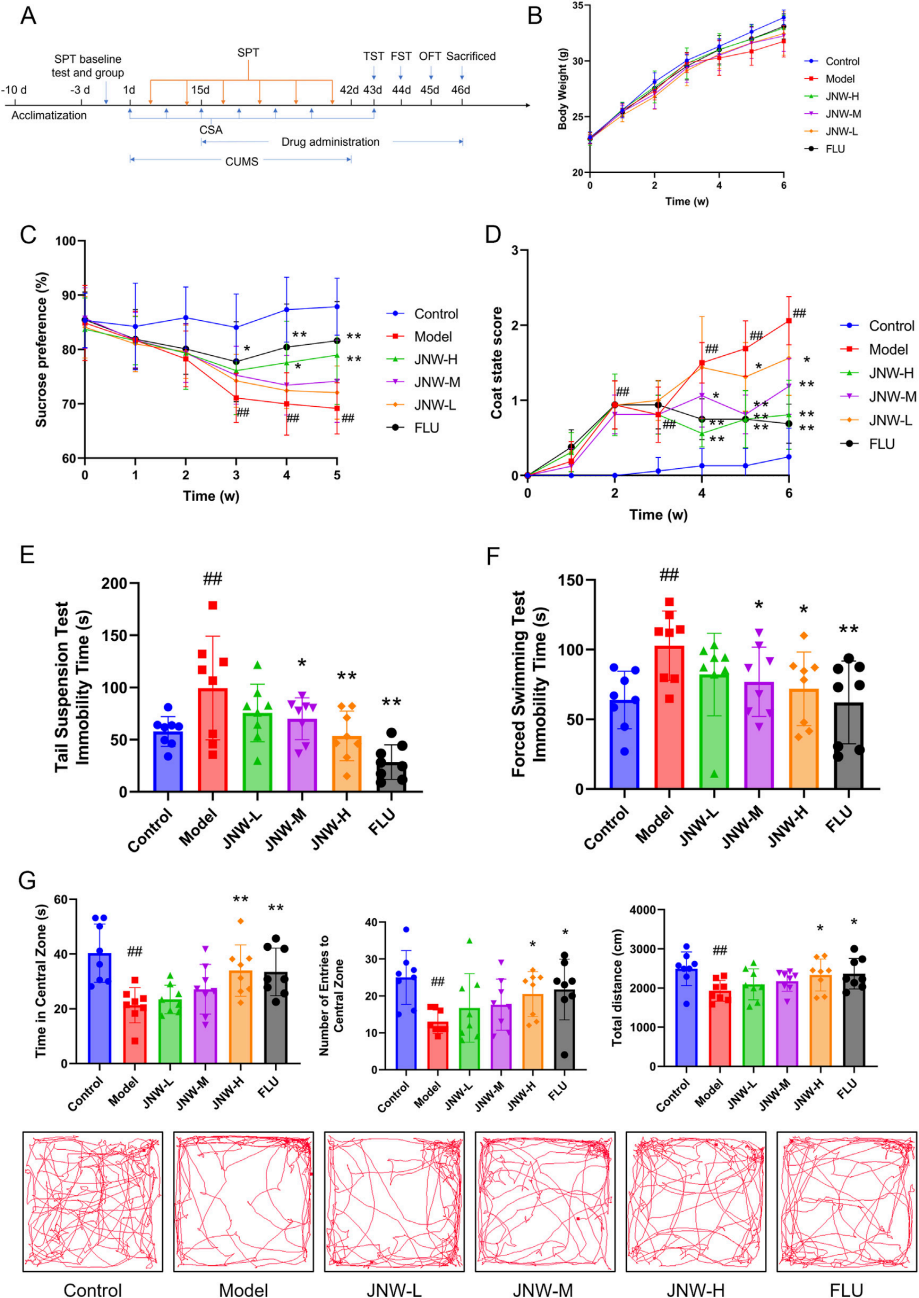
JNW improve depression-like behavior in CUMS induced mice. Mice were intragastric administered water (control and model group), JNW 1.5 (JNW-H), 0.75 (JNW-M), 0.375 (JNW-L) g/kg or fluoxetine 10 mg/kg (FLU) for 32 consecutive days after 2 weeks CUMS exposure. **(A)** Schematic timeline of experimental procedures. **(B)** Body weight of mice during CUMS modeling. **(C)** Sucrose preference of mice during CUMS modeling. **(D)** Coat state score of mice during CUMS modeling. **(E)** Immobility time of mice in TST after CUMS modeling. **(F)** Immobility time of mice in FST after CUMS modeling. **(G)** Time spent in the central zone, number of entries to central zone and movement distance in OFT after CUMS modeling. All values are presented as means ± SEM (*n* = 8). Compared with the control group, ^##^P < 0.01; compared with model group, ^*^P < 0.05, ^**^P < 0.01.

The flowchart of the experimental protocol is presented in Figure 1A. After a week of acclimatization, mice were allocated into six groups (*n* = 8) based on the sucrose preference test (SPT) baseline. Briefly, the mice were first provided with two bottles of 2% sucrose solution for a period of 24 h, and then one bottle was replaced with fresh water, while the other remained with 2% sucrose solution, for another 24 h. Following this training phase, the mice underwent a 12-h fasting period and were then given one bottle of 2% sucrose solution and another of fresh water for an additional 24 h. The position of the two bottles was exchanged every 12 h. Sucrose preference was calculated using the following formula: Sucrose preference (%) = [sucrose solution intake/(sucrose solution intake + water intake)] × 100%.

Every four mice from the control group were housed together in each cage, whereas other mice were housed individually. After 2 weeks chronic unpredictable mild stress (CUMS) exposure, from day 15 to day 46, drug administration is conducted between 8:00 a.m. and 9:00 a.m. once a day for 32 consecutive days. JNW-L, JNW-M, JNW-H group were intragastric administered JNW 0.375, 0.75 and 1.5 g/kg respectively, FLU group were intragastric administered fluoxetine 10 mg/kg, while control and model group were intragastric administered water. The dosage of JNW-L is determined based on the human equivalent dose conversion and the administration duration is determined according to clinical experience. And the dosage of fluoxetine was selected according to the reference literature (Lin et al., 2023), which was equivalent to the maximum recommended human dose.

### 2.5 CUMS procedure

The CUMS procedure was executed in accordance with previously described (Nollet, 2021) with minor modification. In brief, all mice except control group were exposed 1–2 the following stimuli every day for 6 weeks (from day 1 to day 42): food deprivation for 12 h, water deprivation for 12 h, water cage (∼1 cm of water in empty cage) for 4 h, tilted cage for 12 h, dampened sawdust for 12 h, rat feces (∼60 mL of rat sawdust placed inside the cage) for 2 h, pair housing (housing with another mice randomly) for 2 h, cage changing (residing in a cage that was previously inhabited by a different individual) for 12 h, body restraining (confined within a 50 mL conical tube, maintaining a steady respiration) for 2 h, cold water (swimming in 10°C water) for 5 min, dampened sawdust for 12 h. In order to ensure unpredictability and reduce the impact to SPT and coat state assessment (CSA), the stimuli were administered in a semi-random schedule throughout the week, as outlined below (Table 1).

**TABLE 1:** Stimuli schedule during a week.

Day of Week	Day	Night
Monday	Body restraining (9:00–11:00)	Cold water (15:00–15:05)
Tuesday	Pair housing (9:00–11:00)	Dampened sawdust (21:00–9:00)
Wednesday	Water cage (9:00–13:00)	Rat feces (16:00–18:00)
Thursday	Cold water (9:00–9:05)	-
Friday	Pair housing (9:00–11:00)	Body restraining (16:00–18:00)
Saturday	Water deprivation (9:00–21:00)	Tilted cage (21:00–9:00)
Sunday	Food deprivation (9:00–21:00)	Cage changing (21:00–9:00)

### 2.6 Behavioral tests

#### 2.6.1 SPT

SPT was carried out from every Thursday (day 4, 11, 18, 25, 32, 39) 8:00 a.m. to Friday 8:00 a.m. and performed after 12 h fasting. All mice were provided with a bottle of 2% sucrose solution and another bottle of fresh water for 24 h. Every 12 h, the positions of the two bottles were swapped. Sucrose preference was calculated as previously described.

#### 2.6.2 CSA

CSA was carried out every Monday (day 1, 8, 15, 22, 29, 36, 43) at 8:00 a.m. before stimulating. Evaluate the coat condition in the following seven regions of the body: head, neck, back, abdomen, tail, forepaws, and hindpaws. Assign a score to the coat state in each area as follows: 0 (good) for smooth and shiny fur, with any tousled or spiky patches; 0.5 (moderate) for slightly fluffy fur with a few spiky patches; 1 (bad) for unkempt fluffy fur with minor staining. Add up the scores from all seven areas to calculate the total score.

#### 2.6.3 Tail suspension test (TST)

TST was performed 30 min after drug administration on day 43, between 9:00 a.m. and 11:00 a.m. Mice were suspended 25 cm above the table by securing their tail tips with tape to an iron stand and separated by partitions to prevent them from seeing each other. The experiment lasted for a total of 6 min in an undisturbed environment, and the duration of immobility during the final 5 min of this period was recorded.

#### 2.6.4 Forced swimming test (FST)

FST was conducted 30 min after drug administration on day 44, between 9:00 a.m. and 11:00 a.m. A clear cylindrical container, measuring 20 cm in diameter and 30 cm in height, was filled with water (25°C ± 2°C) to a level of approximately 20 cm. Mice were singly placed into the cylinder and forced to swim for a total duration of 6 min. And the duration of immobility in the last 5 min was recorded.

#### 2.6.5 Open field test (OFT)

The OFT was conducted 30 min after drug administration on day 45 morning. The OFT apparatus (Noldus, Netherlands) was made of black polyvinyl chloride (50 × 50 × 40 cm) and the floor was divided into a central zone (25 × 25 cm) and a peripheral zone. The OFT was carried out in an undisturbed environment with an indirect artificial light source (∼50 lux) and the mice were acclimatized in their home cage for 1 h. Each mouse was placed individually in the middle of the central area and the behavior was recorded for 5 min. The duration spent in central zone, number of entries to central zone and the total distance moved were documented. The OFT device was cleaned with 75% ethanol and paper towel after each trial to remove the trace scents and other matter.

### 2.7 Sample collections

Mice were anesthetized with 60 mg/kg pentobarbital sodium by intraperitoneal injection, 30 min after the last intragastric administration. Blood samples were collected and serum was separated by centrifugation at 800 *g* for 10 min. Then mice were rapidly decapitated, and the hippocampus and hypothalamus were extracted on ice, immediately frozen in liquid nitrogen, and then stored at −80°C.

### 2.8 ELISA

Left hippocampus and hypothalamus were weighted and homogenized in nine volumes of phosphate buffer solution (PBS) at 4°C. The supernatant was separated through centrifugation at 800 *g* for 10 min. The concentrations of 5-HT, NE, IL-1β, IL-6, TNF-α in both the serum and hippocampus, ACTH, CORT in the serum, and CRH in the hypothalamus were determined by ELISA.

### 2.9 Quantitative reverse transcription polymerase chain reaction (qRT-PCR)

From each group, three right hippocampi were randomly chosen for further analysis. Total RNA was isolated and the first cDNA was reverse transcripted. qRT-PCR was run on an Archimed X4 instrument (Rocgene, Beijing, China) under the specified conditions: 95°C for 2 min, then 40 cycles at 95°C for 5 s and 60°C for 34 s. The primer sequences are detailed in Table 2. The 2^−ΔΔCT^ method was employed to quantify gene expression of IL-1β, IL-6, TNF-α, Bax, Bcl-2, NLRP3 and Caspase-1, with expression of GAPDH used for normalization.

**TABLE 2:** Primer information.

Gene		Primer sequences (5′-3′)	Product length (bp)
GADPH	Forward	GCC​CAG​AAC​ATC​ATC​CCT​GCA​T	188
	Reverse	GCC​TGC​TTC​ACC​ACC​TTC​TTG​A	
IL-1β	Forward	GCA​ACG​ACA​AAA​TAC​CTG​TGG​CC	205
	Reverse	CAG​TTG​GGG​AAC​TCT​GCA​GAC​TC	
IL-6	Forward	GGA​TAC​CAC​TCC​CAA​CAG​ACC​TG	278
	Reverse	TGT​TCT​TCA​TGT​ACT​CCA​GGT​AGC​T	
TNF-α	Forward	GGT​GCC​TAT​GTC​TCA​GCC​TCT​TC	161
	Reverse	TGA​TCT​GAG​TGT​GAG​GGT​CTG​GG	
Bax	Forward	GTT​TCA​TCC​AGG​ATC​GAG​CAG​GG	169
	Reverse	GTG​TCC​ACG​TCA​GCA​ATC​ATC​CT	
Bcl-2	Forward	ACT​CTT​CAG​GGA​TGG​GGT​GAA​CT	132
	Reverse	TAC​TCA​GTC​ATC​CAC​AGG​GCG​AT	
NLRP3	Forward	GGT​TGG​TGA​ATT​CCG​GCC​TTA​CT	272
	Reverse	ATC​ATT​GTT​GCC​CAG​GTT​CAG​CT	
Caspase 1	Forward	TGA​CTG​GGA​CCC​TCA​AGT​TTT​GC	212
	Reverse	GGT​ATA​CCC​CAG​ATC​CTC​CAG​CA	

### 2.10 Western blot

Three right hippocampi in each group were randomly selected. The total protein was extracted with RIPA lysis buffer and protein concentration was determined by BCA method. The protein extracts underwent SDS-PAGE and were subsequently electro-transferred onto nitrocellulose membranes. The membranes were then blocked using 5% non-fat milk powder for 2 h. The membranes were incubated with the primary antibody, diluted to a ratio of 1:1000, overnight at 4°C and then incubated with secondary antibody conjugated to HRP. The antigen–antibody complexes were detected using an ECL reagent. The intensities of the Western blotting bands corresponding to NLRP3, Caspase-1, NF-κB p65, IκBα, p-IκBα, Bax and Bcl-2 were normalized to β-actin, which served as a loading control.

### 2.11 Statistical analysis

Data were presented in the form of mean ± S.E.M. Statistical analysis was carried out using SPSS 26.0. The data of body weight and SPT was analyzed by two-way repeated measures analysis of variance (ANOVA), the data of CSA was analyzed by generalized estimating equation, the data of number of entries to central zone in OFT was analyzed by Kruskal–Wallis one-factor analysis of rank variance, and other data was analyzed by one-way ANOVA. The *post hoc* analysis conducted by LSD tests. The study established a statistical significance threshold with a p-value of less than 0.05.

## 3 Results

### 3.1 JNW improve depression-like behavior in mice induced by CUMS

During the process of CUMS modeling, the mice progressively exhibited the symptoms that decrease in locomotor activity and insensitive to external stimuli. Body weight of model group was lower compared to control group, while JNW groups recovered to a certain extent. However, there is no significant differences between all groups (F(5,42) = 1.664, P > 0.05) (Figure 1B).

Decrease in sucrose consumption reflected the symptom of anhedonia in CUMS mice (Pothion et al., 2004). As shown in Figure 1C, it was found that the sucrose preference showed a significant change between the groups during the experiment (F(5,42) = 4.728, P < 0.01, partial η^2^ = 0.360). Intergroup analysis revealed that starting from the third week of modeling, the sucrose preference in the model group was significantly lower than that in the normal group (3w: P < 0.01, Cohen’s d = 2.091, 4w: P < 0.01, Cohen’s d = 2.793, 5w: P < 0.01, Cohen’s d = 2.812). However, JNW was observed to improve the sucrose preference of CUMS mice, and the JNW-H group demonstrated a significant difference compared to the model group at 4w and 5w with strong effect (4w: P < 0.05, Cohen’s d = 1.220, 5w: P < 0.01, Cohen’s d = 1.473). The fluoxetine group showed a statistically significant difference from the model group starting from the third week (3w: P < 0.05, Cohen’s d = 1.078, 4w: P < 0.01, Cohen’s d = 1.683, 5w: P < 0.01, Cohen’s d = 1.870). Although its sucrose preference was higher than that of the JNW-H group at the same time point, there was no significant difference between the two groups (P > 0.05).

The coat state, determined by the frequency and intensity of grooming behavior, serves as an indicator of the mice’s motivation for self-centered activities (Burstein and Doron, 2018). As the CUMS modeling progressed, the coat of the mice in the model group gradually became notably disheveled, fluffy, and stained (Figure 1D). There was a statistically significant difference between the groups during experimental period (z = 2.403, P < 0.05). Beginning from the second week post-modeling, the CSA score of model group showed a statistically significant difference (2w: P < 0.01, Cohen’s d = 3.099, 3w: P < 0.01, Cohen’s d = 2.658, 4w: P < 0.01, Cohen’s d = 3.628, 5w: P < 0.01, Cohen’s d = 4.406, 6w: P < 0.01, Cohen’s d = 4.664) compared to normal group. JNW demonstrates a time- and dose-dependent recovery in CSA. The JNW-H group exhibited significant improvement starting from the fourth week (4w: P < 0.01, Cohen’s d = 2.474, 5w: P < 0.01, Cohen’s d = 2.644, 6w: P < 0.01, Cohen’s d = 3.216), as did the JNW-M group (4w: P < 0.05, Cohen’s d = 1.155, 5w: P < 0.01, Cohen’s d = 2.468, 6w: P < 0.01, Cohen’s d = 2.251). The JNW-L group also exhibited significant improvement starting from the fifth week (5w: P < 0.05, Cohen’s d = 1.058, 6w: P < 0.05, Cohen’s d = 1.287). Although fluoxetine group showed a greater reduction in CSA score, there was no significant difference compared to the JNW-H group (P > 0.05).

Both TST and FST are extensively utilized in antidepressant screening, due to the immobile time is indicative of behavioral despair of the mice (Yan et al., 2010). As shown in Figures 1E,F, following the administration of JNW, a dose-dependent reduction in immobility time was observed in both the TST (F(5,42) = 5.837, P < 0.01, partial η^2^ = 0.410) and FST (F(5,42) = 2.594, P < 0.05, partial η^2^ = 0.236). The JNW-M (P < 0.05, Cohen’s d = 1.052) and JNW-H (P < 0.01, Cohen’s d = 1.642) groups exhibited statistically significant differences compared to the model group in TST, and the JNW-H groups showed a significant difference compared to model group in FST (P < 0.05, Cohen’s d = 1.181). No statistically significant difference was observed between the JNW-H group and the fluoxetine group (P > 0.05).

In OFT, rodents exhibit thigmotaxis, yet they also possess a desire to explore the central area of the chamber; however, this exploratory behavior may be inhibited in states of depression and anxiety (Kraeuter et al., 2019). CUMS led to a noticeable decrease in the number of entries into the central zone (H = 13.455, P < 0.05, partial η^2^ = 0.201), the duration spent in the central zone (F(5,42) = 5.871, P < 0.01, partial η^2^ = 0.411) and the overall movement distance (F(5,42) = 2.503, P < 0.05, partial η^2^ = 0.230). Nonetheless, JNW was able to reverse these alterations, with high-dose groups demonstrating a statistically significant difference (P < 0.05, Cohen’s d = 1.464; P < 0.01, Cohen’s d = 1.498; P < 0.05, Cohen’s d = 1.101; corresponding to the above three indicators, respectively) when compared to the model group and exhibiting no statistically significant difference compared to the fluoxetine group (P > 0.05) (Figure 1G).

### 3.2 JNW suppress the CUMS-induced pro-inflammation cytokine production

**FIGURE 2:**
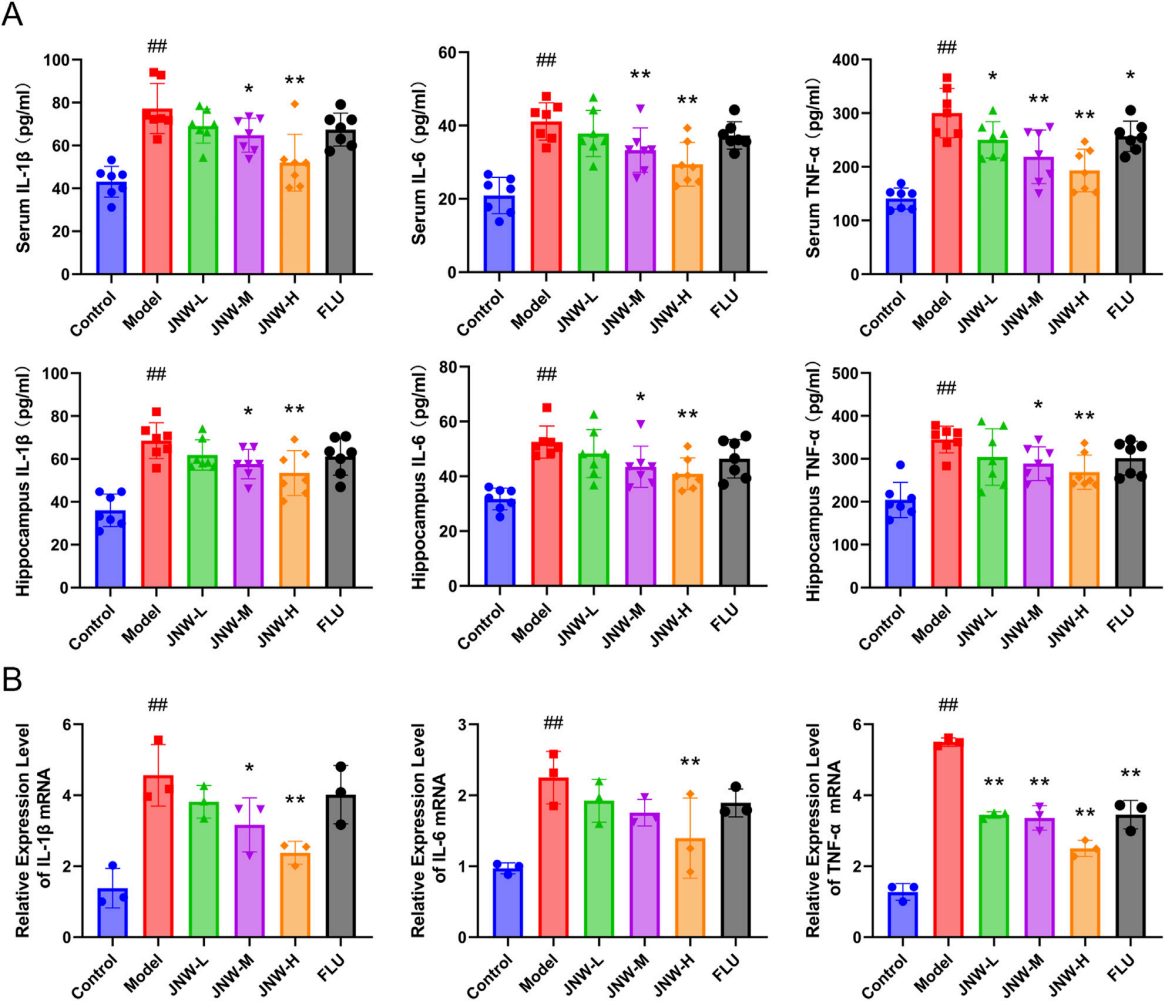
JNW suppress the CUMS-induced pro-inflammation cytokine production. Serum and hippocampus were collected from the mice of control, model, JNW-L (0.375 g/kg), JNW-M (0.75 g/kg), JNW-H (1.5 g/kg) and FLU (10 mg/kg) group. **(A)** The concentration of IL-1β, IL-6, and TNF-α both in serum and hippocampus were detected by ELISA. All values are presented as means ± SEM (*n* = 7). **(B)** The mRNA expression of IL-1β, IL-6, and TNF-α in hippocampus were measured by qRT-PCR. mRNA level was normalized to and expressed as folds of β-actin. All values are presented as means ± SEM (*n* = 3). Compared with the control group, ^##^P < 0.01; compared with model group, ^*^P < 0.05, ^**^P < 0.01.

A growing amount of data indicates that the overproduction of pro-inflammatory cytokines, such as IL-1β, IL-6, and TNF-α, by immune cells within the brain, plays an important role in the onset and progression of depression (Beurel et al., 2020). Consistent with previous reported findings, it was found that the concentrations of IL-1β, IL-6, and TNF-α in both serum (IL-1β: F(5,36) = 11.896, P < 0.01, partial η^2^ = 0.623; IL-6: F(5,36) = 12.681, P < 0.01, partial η^2^ = 0.638; TNF-α: F(5,36) = 15.240, P < 0.01, partial η^2^ = 0.679) and the hippocampus (IL-1β: F(5,42) = 12.874, P < 0.01, partial η^2^ = 0.641; IL-6: F(5,36) = 8.143, P < 0.01, partial η^2^ = 0.531; TNF-α: F(5,36) = 7.922, P < 0.01, partial η^2^ = 0.524) were dramatically elevated in CUMS model group. Interestingly, JNW could suppress these three pro-inflammation cytokines production in a dose-dependent manner (Figure 2A). When compared to the model group, both the JNW-H group (IL-1β in serum: P < 0.01, Cohen’s d = 2.641, in the hippocampus: P < 0.01, Cohen’s d = 1.834; IL-6 in serum: P < 0.01, Cohen’s d = 2.155, in the hippocampus: P < 0.01, Cohen’s d = 1.742; TNF-α in serum: P < 0.01, Cohen’s d = 2.843, in the hippocampus: P < 0.01, Cohen’s d = 1.719) and JNW-M group (IL-1β in serum: P < 0.05, Cohen’s d = 1.302, in the hippocampus: P < 0.01, Cohen’s d = 1.323; IL-6 in serum: P < 0.01, Cohen’s d = 1.443, in the hippocampus: P < 0.05, Cohen’s d = 1.354; TNF-α in serum: P < 0.01, Cohen’s d = 2.162, in the hippocampus: P < 0.05, Cohen’s d = 1.271) exhibited statistically significant differences, whether in the serum or in the hippocampus. While the fluoxetine group exhibited a significant difference only in terms of suppressing the level of serum TNF-α (P < 0.05, Cohen’s d = 1.150).

Furthermore, qRT-PCR was employed to further investigate the gene transcript levels of the pro-inflammatory cytokines. In line with the expected, JNW downregulated IL-1β, IL-6, and TNF-α mRNA expression (IL-1β: F(5,12) = 9.453, P < 0.01, partial η^2^ = 0.798; IL-6: F(5,12) = 5.843, P < 0.01, partial η^2^ = 0.709; TNF-α: F(5,12) = 84.545, P < 0.01, partial η^2^ = 0.972) (Figure 2B). When compared to the model group, high-dose JNW significantly reduced the expression of IL-1β (P < 0.01, Cohen’s d = 3.303), IL-6 (P < 0.01, Cohen’s d = 2.639), and TNF-α (P < 0.01, Cohen’s d = 11.479), medium-dose JNW significantly decreased the expression of IL-1β (P < 0.05, Cohen’s d = 2.115) and TNF-α (P < 0.01, Cohen’s d = 8.175), while the low-dose JNW treatment significantly suppressed the expression of TNF-α only (P < 0.05, Cohen’s d = 7.857). Fluoxetine exhibited an inhibitory effect on the transcription of TNF-α mRNA in the hippocampus (P < 0.01, Cohen’s d = 7.831), but its suppressive effect on the transcription of all those inflammatory cytokines was weaker than that of JNW-H.

### 3.3 JNW reduce the level of HPA axis hormone

**FIGURE 3:**
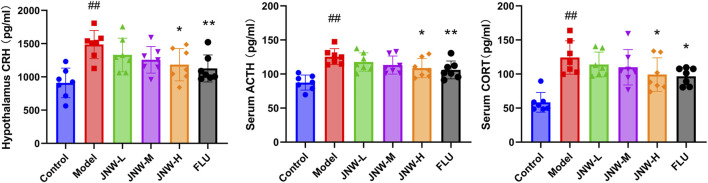
JNW reduce the level of hypothalamic-pituitary-adrenal (HPA) axis hormone. Serum and hypothalamus were collected from the mice of control, model, JNW-L (0.375 g/kg), JNW-M (0.75 g/kg), JNW-H (1.5 g/kg) and FLU (10 mg/kg) group and the level of CRH, ACTH and CORT were detected by ELISA. All values are presented as means ± SEM (*n* = 7). Compared with the control group, ^##^P < 0.01; compared with model group, ^*^P < 0.05, ^**^P < 0.01.

A substantial amount of clinical and basic research has indicated significant associations between alterations in HPA axis hormone dynamics and depression (Menke, 2024). As shown in Figure 3, the statistically significantly elevated level of CRH in the hypothalamus (F(5,36) = 5.371, P < 0.01, partial η^2^ = 0.427), as well as ACTH (F(5,36) = 7.106, P < 0.01, partial η^2^ = 0.497) and CORT (F(5,36) = 8.463, P < 0.01, partial η^2^ = 0.540) in the serum, reflected an altered HPA axis response to CUMS. JNW has a certain recovery effect on the content of CRH, ACTH and CORT in CUMS model mice. In particular, the JNW-H group exhibited significantly decreased levels of CRH (P < 0.05, Cohen’s d = 1.357), ACTH (P < 0.05, Cohen’s d = 1.308), and CORT (P < 0.05, Cohen’s d = 1.205) compared to the model group. Meanwhile, the potency of JNW-H is comparable to that of fluoxetine, with no statistically significant difference between the two (P > 0.05).

### 3.4 JNW increase the level of monoamine neurotransmitter in both serum and hippocampus

**FIGURE 4:**
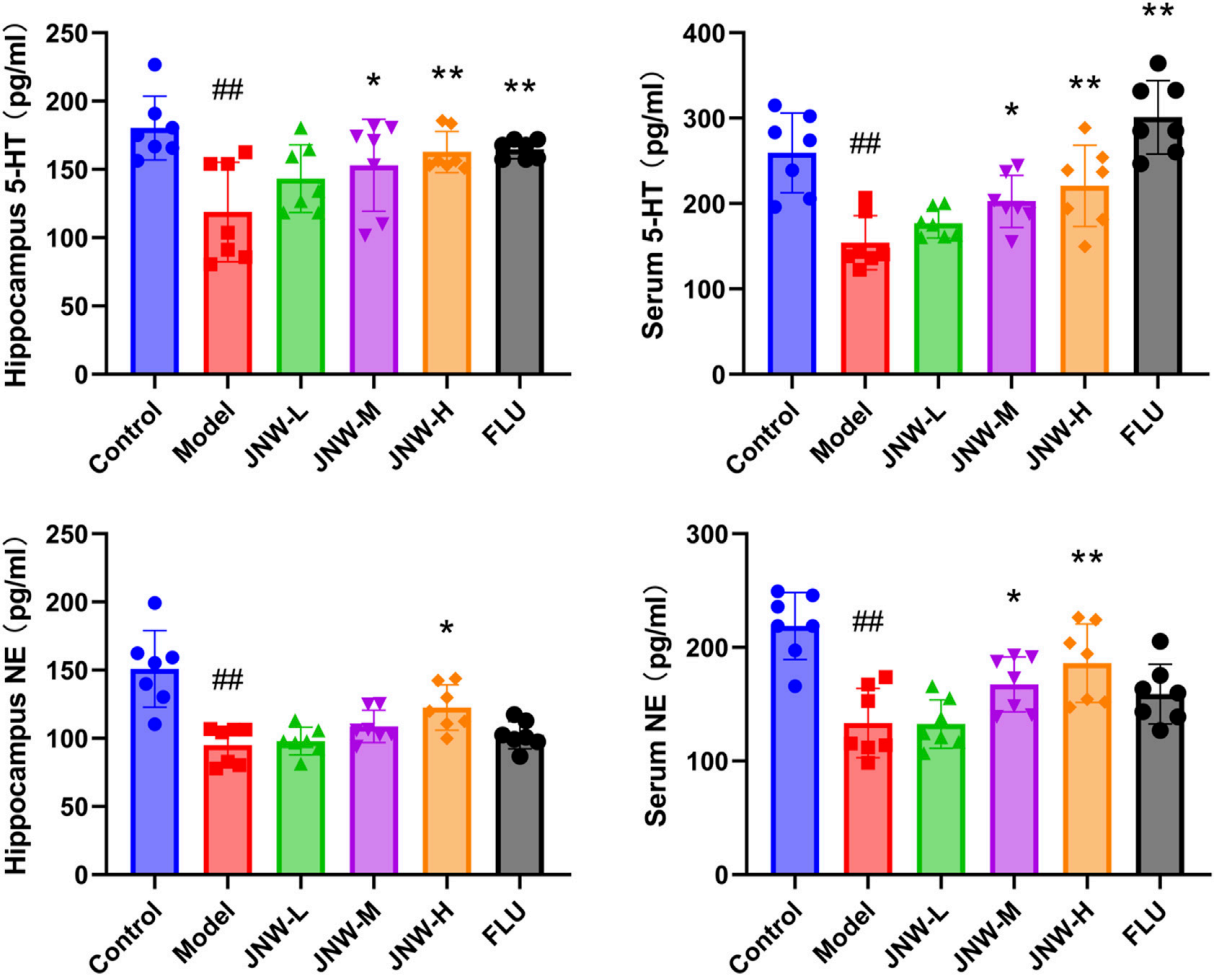
JNW increase the level of monoamine neurotransmitter in both serum and hippocampus. Serum and hippocampus were collected from the mice of control, model, JNW-L (0.375 g/kg), JNW-M (0.75 g/kg), JNW-H (1.5 g/kg) and FLU (10 mg/kg) group and the level of 5-HT and NE were detected by ELISA. All values are presented as means ± SEM (*n* = 7). Compared with the control group, ^##^P < 0.01; compared with model group, ^*^P < 0.05, ^**^P < 0.01.

The widely accepted hypothesis is that deficiencies or imbalances in monoamine neurotransmitters, such as 5-HT, NE, and dopamine (DA), is a crucial factor contributing to the progression of depression (Perez-Caballero et al., 2019). In the present research (Figure 4), it was found that the level of 5-HT (Serum: F(5,36) = 14.473, P < 0.01, partial η^2^ = 0.668; hippocampus: F(5,36) = 4.812, P < 0.01, partial η^2^ = 0.401) and NE (Serum: F(5,36) = 9.643, P < 0.01, partial η^2^ = 0.573; hippocampus: F(5,36) = 11.615, P < 0.01, partial η^2^ = 0.617) in both serum and hippocampus was dramatically decreased in CUMS induced depression-like mice, aligning with previous findings. The contents of 5-HT in both hippocampus and serum exhibited a dose-dependent increase across all JNW treatment groups. And significant differences were noted between the high-dose (serum: P < 0.01, Cohen’s d = 1.769; hippocampus: P < 0.01, Cohen’s d = 1.721) and medium-dose groups (serum: P < 0.05, Cohen’s d = 1.290; hippocampus: P < 0.05, Cohen’s d = 1.343) compared to the model group. Meanwhile, JNW exerted a discernible effect on elevating NE concentrations in both the hippocampus and serum. Specifically, the medium-dose JNW significantly enhanced NE levels in the serum (P < 0.05, Cohen’s d = 1.213), whereas the high-dose led to a substantial increase in NE levels in both the serum and hippocampus (serum: P < 0.01, Cohen’s d = 1.877; hippocampus: P < 0.01, Cohen’s d = 1.695). This dose-dependent discrepancy in elevating NE levels between serum and hippocampal tissues may be due to differences in the tissue origins of NE synthesis, as well as the metabolic and distribution profiles of JNW components. Fluoxetine, as a selective serotonin reuptake inhibitor, only increased 5-HT levels in the serum and hippocampus, whereas it did not exert a significant influence on NE levels.

### 3.5 JNW influence the expression of Bax and Bcl-2 in hippocampus of CUMS-induced mice

**FIGURE 5:**
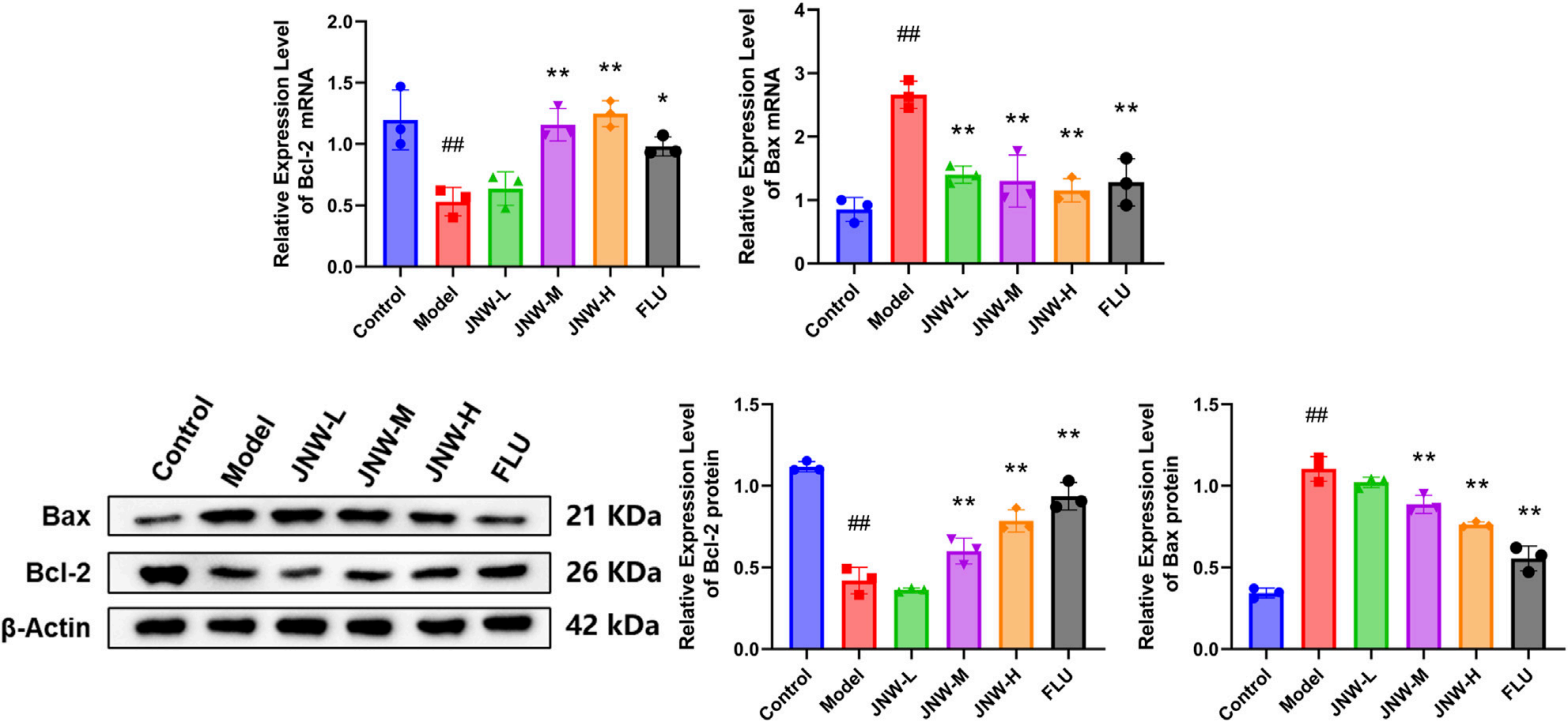
JNW influence the Bax and Bcl-2 expression in hippocampus of CUMS-induced mice. Hippocampus were collected from the mice of control, model, JNW-L (0.375 g/kg), JNW-M (0.75 g/kg), JNW-H (1.5 g/kg) and FLU (10 mg/kg) group. The mRNA expression of Bax and Bcl-2 were detected by qRT-PCR and the protein content were measured by Western blot. Both mRNA and protein level were normalized to and expressed as folds of β-actin. All values are presented as means ± SEM (*n* = 3). Compared with the control group, ^##^P < 0.01; compared with model group, ^*^P < 0.05, ^**^P < 0.01.

Chronic stress has been demonstrated to elevate the vulnerability of specific neuronal populations to undergoing apoptosis (McKernan et al., 2009). As Bax and Bcl-2 are widely recognized as common markers of apoptosis, this research endeavors to evaluate the influence of JNW on apoptosis by analyzing these markers. As shown in Figure 5, the expression of Bcl-2 in the hippocampus was downregulated at both of mRNA (F(5,12) = 13.288, P < 0.01, partial η^2^ = 0.847) and protein (F(5,12) = 89.006, P < 0.01, partial η^2^ = 0.974) levels due to CUMS, while the expression of Bax was upregulated at both of mRNA (F(5,12) = 16.146, P < 0.01, partial η^2^ = 0.871) and protein levels (F(5,12) = 61.514, P < 0.01, partial η^2^ = 0.962). It was discovered that high-dose JNW significantly increased the expression levels of Bcl-2 mRNA (P < 0.01, Cohen’s d = 4.937) and protein (P < 0.01, Cohen’s d = 6.466), while suppressing the expression of Bax mRNA (P < 0.01, Cohen’s d = 5.580) and protein (P < 0.01, Cohen’s d = 5.591), as compared to the model group. And medium-dose JNW also increased the expression levels of Bcl-2 mRNA (P < 0.01, Cohen’s d = 4.317) and protein (P < 0.01, Cohen’s d = 4.111) and suppressed the expression of Bax mRNA (P < 0.01, Cohen’s d = 5.037) and protein (P < 0.01, Cohen’s d = 2.768). These results indicated that JNW may exert a protective effect on the apoptosis in hippocampus of CUMS-induced mice.

### 3.6 JNW inhibited NF-κB/NLRP3 pathway activation in hippocampus of CUMS-induced mice

**FIGURE 6:**
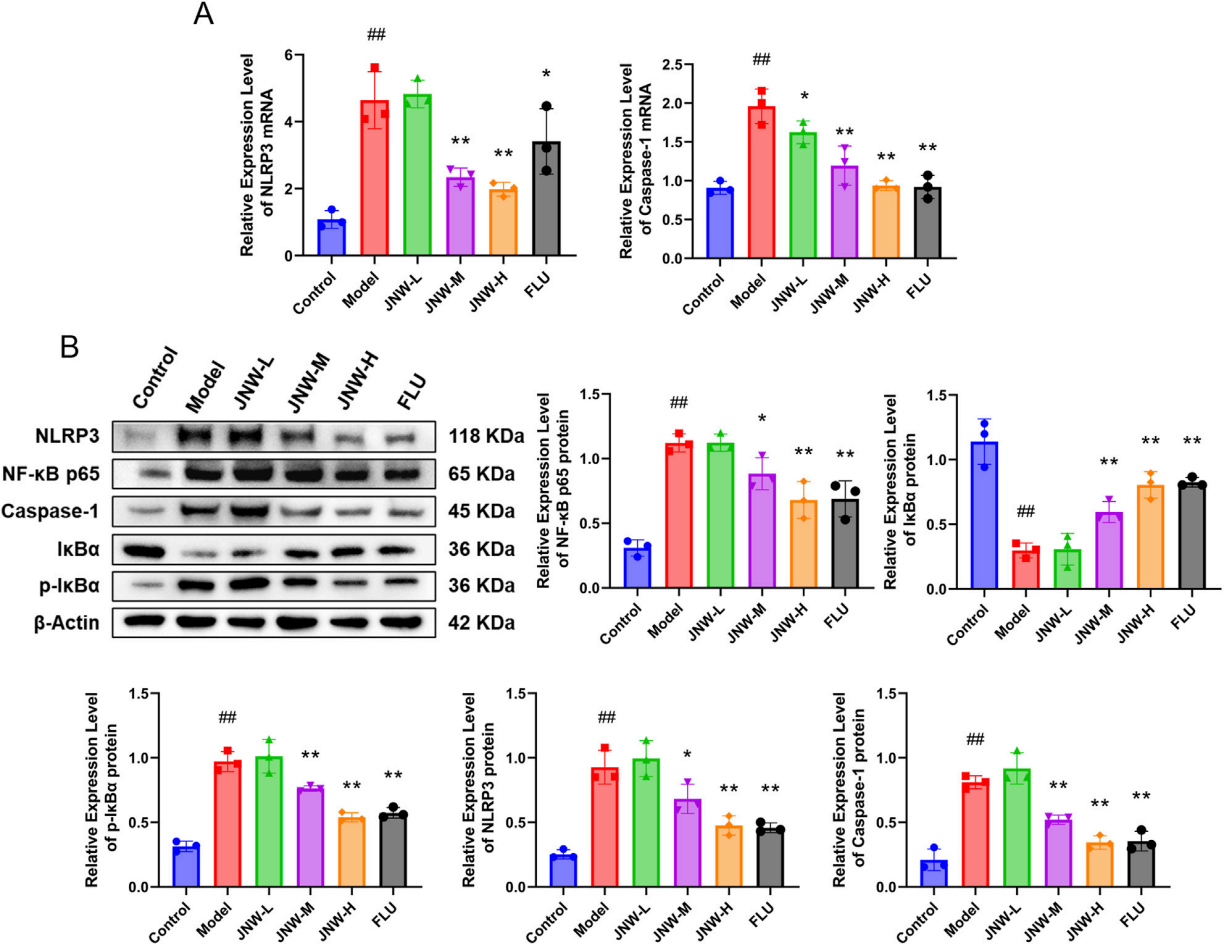
JNW attenuated NF-κB/NLRP3 pathway activation in hippocampus of CUMS-induced mice. Hippocampus were collected from the mice of control, model, JNW-L (0.375 g/kg), JNW-M (0.75 g/kg), JNW-H (1.5 g/kg) and FLU (10 mg/kg) group. **(A)** mRNA expression of NLRP3 and Caspase-1. **(B)** The protein content of NF-κB p65, p-IκBα, IκBα, NLRP3 and Caspase-1. The mRNA expression was detected by qRT-PCR and the protein content was measured by Western blot. Both mRNA and protein level were normalized to and expressed as folds of β-actin. All values are presented as means ± SEM (*n* = 3). Compared with the control group, ^##^P < 0.01; compared with model group, ^*^P < 0.05, ^**^P < 0.01.

NLRP3 inflammasome is an intracellular multiprotein complex that has been identified as a critical factor in neuroinflammation. Additionally, the activation of the NF-κB signaling pathway is crucial for the expression of NLRP3, which in turn promotes the activation of the NLRP3 inflammasome. As shown in Figure 6, it was showed that CUMS induce to a marked increase in the mRNA and protein expression of NLRP3 (mRNA: F(5,12) = 20.016, P < 0.01, partial η^2^ = 0.893; protein: F(5,12) = 26.106, P < 0.01, partial η^2^ = 0.916) and Caspase-1 (mRNA: F(5,12) = 20.762, P < 0.01, partial η^2^ = 0.896; protein: F(5,12) = 42.134, P < 0.01, partial η^2^ = 0.946) in hippocampus. Concurrently, there was a notable decrease in IκBα content (F(5,12) = 28.546, P < 0.01, partial η^2^ = 0.922), whereas p-IκBα (F(5,12) = 47.134, P < 0.01, partial η^2^ = 0.952) and NF-κB p65 (F(5,12) = 25.283, P < 0.01, partial η^2^ = 0.913) content underwent significant elevations. These results suggested that the NF-κB/NLRP3 pathway was activated by CUMS and may play a role in contributing to depression-like behaviors observed in the model mice. However, JNW exhibited a dose-dependent inhibitory effect on the activation of the NF-κB/NLRP3 pathway. It was found that the mRNA expression levels of NLRP3 (JNW-H: P < 0.01, Cohen’s d = 4.564; JNW-M: P < 0.01, Cohen’s d = 3.947) and Caspase-1 (JNW-H: P < 0.01, Cohen’s d = 6.108; JNW-M: P < 0.01, Cohen’s d = 4.576) were significantly suppressed, while IκBα content (JNW-H: P < 0.01, Cohen’s d = 4.760; JNW-M: P < 0.01, Cohen’s d = 2.789) was increased and the phosphorylation of IκBα (JNW-H: P < 0.01, Cohen’s d = 6.344; JNW-M: P < 0.01, Cohen’s d = 3.072), as well as the protein levels of NF-κB p65 (JNW-H: P < 0.01, Cohen’s d = 4.131; JNW-M: P < 0.05, Cohen’s d = 2.225), NLRP3 (JNW-H: P < 0.01, Cohen’s d = 4.595; JNW-M: P < 0.05, Cohen’s d = 2.501), and Caspase-1 (JNW-H: P < 0.01, Cohen’s d = 6.198; JNW-M: P < 0.01, Cohen’s d = 3.859), were all reduced in the JNW-H and JNW-M groups compared to the model group. Meanwhile, it was also observed that fluoxetine exhibited similar effects, and no significant difference was found between JNW and fluoxetine (P > 0.05).

## 4 Discussions

Depression is a prevalent mentally disease around the world, which is not only harmful to individuals, but also have an increasing impact on the economic burden. However, due to its intricate and elusive etiology, the therapy for depression remains clinically unsatisfactory despite the use of certain drugs (tricyclic antidepressants, monoamine oxidase inhibitors, selective monoamine reuptake inhibitors, NMDAR antagonist, etc.) for decades (Marwaha et al., 2023). Increasing attention is being given to alternative therapy methods. A substantial number of preclinical and clinical researches have confirmed the antidepressant effects of traditional medicine, including *Hypericum perforatum* and *saffron crocus* in European, as well as *Bupleurum chinense* and *Ginkgo biloba* in East Asia (Dobrek and Głowacka, 2023).

In the TCM theory, mental disorders like depression and anxiety are frequently associated with ‘deficiency of qi and blood’ and ‘kidney essence deficiency.’ According this theory, JNW has been employed to treat depression and anxiety, and has demonstrated proven effectiveness. In previous study, totally 86 compounds, containing 19 terpenoids, 17 alkaloids, 8 phenylpropanoids, 14 flavonoids, 3 phenols and so on, were identified in JNW. And it was found that JNW effectively alleviated anxiety-like behaviors induced by chronic restraint stress in mice and potentially improved their neural functions by regulating their intestinal microbiota (Pan et al., 2024). However, additional investigation is necessary to comprehensively understand the antidepressant effect and mechanism of JNW.

It is widely recognized that stress plays a pivotal role in the onset and progression of depression. Given this consideration, the CUMS model, which could induce behavioral impairments such as anhedonia and behavioral despair, along with alterations in specific endocrine and neural parameters (Antoniuk et al., 2019), has been employed to investigate the antidepressant properties of JNW. As expected, it was found that the sucrose preference and coat state was progressively deteriorated during CUMS stimulation in model group. However, JNW could significantly improve these parameters, indicating that it could alleviate the anhedonia and boost the motivation towards self-centered activities in depressive model mice. Meanwhile, JNW demonstrated a notable ability to shorten the period of immobility in both TST and FST, suggesting its effect on improving the behavioral despair in model mice. Additionally, JNW enhanced the model mice’s behavior in an OFT by increasing the frequency of entries into central zone, the duration in the central zone, and the overall movement distance, demonstrating its ameliorative effect on exploratory behavior. Taken together, these findings indicated that JNW exert a beneficial influence on improving depression-like behavior in CUMS-induced mice, partially proved its effect on depression in clinic.

Recently, a growing body of evidence highlights inflammation as a key factor in the development of stress-induced depression (Cui et al., 2024). In depression, pro-inflammatory cytokines such as IL-1β, IL-6 and TNF-α play a distinct role, not merely facilitating immune responses but also participating in the regulation of neurochemical, neuroendocrine and neuroplasticity processes. It was found that the levels of pro-inflammation cytokines in peripheral blood and brain tissue are elevated both in depressant model animals and in depressant patients, and the level of pro-inflammation cytokines are associated with the severity of depression (Chang et al., 2024), indicating that pro-inflammation cytokines can not only amplify neuroinflammation, but also directly influence behavioral responses to stress (Réus et al., 2023). Intriguingly, JNW significantly reduced the level of IL-1β, IL-6, and TNF-α in both serum and hippocampus in a dose-dependent manner, and downregulated these cytokines mRNA expression accordingly. These results demonstrated that the effect of JNW on alleviating depression-like behavior in CUMS-induced mice is associated with pro-inflammation cytokines inhibition.

It is worthy to note that emerging evidence reveals an intricate pathophysiological cross-talk between pro-inflammatory cytokines and other neurobiological mechanisms in depression, such as HPA axis hyperactivity, monoamine neurotransmitter dysregulation, and hippocampal apoptosis, indicating that pro-inflammatory cytokines may participate in these pathological progressions.

Stress can be regarded as an evolutionary necessity, serving as an essential response to stimuli that are vital for the survival of any organism (Dhabhar, 2014). In mammals, HPA axis, which functions as a negative feedback mechanism, regulates the physiological responses to stress. However, under the circumstance of depression, the HPA axis negative feedback loop becomes dysfunctional, leading to a sustained increase in glucocorticoids (McEwen and Akil, 2020). Increased pro-inflammatory cytokines and an overactive HPA axis have both been implicated in the pathogenesis of depression (Perrin et al., 2019). On the one hand, pro-inflammation cytokines inhibit glucocorticoids receptor signaling and translocation, leading to hyperactivity of HPA axis and exacerbation of the inflammatory response (Kim et al., 2016). On the other hand, glucocorticoids may also prime the inflammatory response of human hippocampal cells through upregulation of inflammatory pathways (Horowitz et al., 2020). Interestingly, the results demonstrated that JNW decreased the concentrations of ACTH and CORT in the serum and the concentration of CRH in the hypothalamus, indicating that JNW may restore the negative feedback of HPA axis, along with the pro-inflammation cytokines inhibition.

It was also well-established that stress could impact monoamine neurotransmitters system, leading to the induction and exacerbation of depression. Researchers have discovered a functional polymorphism in the promoter region of the serotonin transporter gene that mediates the impact of stressful life events on depression (Caspi et al., 2003). Fang et al. discovered that stress elevates the levels of the acute-phase protein lipopolysaccharide-binding protein, which functions as an endogenous inhibitor of dopamine-β-hydroxylase and aromatic-L-amino-acid-decarboxylase, thereby suppressing monoamine biosynthesis (Fang et al., 2023). Notably, pro-inflammation cytokines and their signaling pathways including p38 mitogen activated protein kinase have significant effects on the metabolism of multiple neurotransmitters such as serotonin and dopamine through impact on their synthesis, release and reuptake via tetrahydrobiopterin and kynurenine pathway (Miller et al., 2013). In current research, the results showed that JNW significantly evaluated the level of 5-HT and NE in both hippocampus and serum, suggesting that monoamine neurotransmitters system may be involved in JNW’s effect on depression, and may be worked together with the inhabitation of pro-inflammation cytokines.

Moreover, the correlation between monoamine neurotransmitters and the HPA axis in depression has been recognized for a long time (Mokrani et al., 1997). It was reported that the elevated cortisol induced by stress increases serotonin uptake by enhancing the expression of the serotonin transporter and could downregulate the availability of serotonin in the synaptic cleft, leading to defective serotonergic neurotransmission in the CNS (Tafet et al., 2001). A dysfunction in 5-HT(1A) receptor activity could be also observed due to a hypersecretion of cortisol (Pitchot et al., 2001). Meanwhile, the dysbalance between CRF1 and CRF2 (two CRH receptors) activation and, consequently, alteration of serotoninergic signaling may result in anxiety and depression, associated with hyperactivity of the HPA axis (Kovács et al., 2025). In contrast, serotonin transporter gene knock-out in mice affects HPA-dependent responses to stress (Lanfumey et al., 2000) and serotonin transporter genotype of rhesus macaques modulates HPA axis output during stress (Sorenson et al., 2013). The above studies indicate that there is a close interaction between monoamine neurotransmitters and the HPA axis, with both systems also engaging in cross-talk with inflammatory cytokines, forming a complex regulatory network. Importantly, JNW effectively ameliorates the disturbances in inflammatory cytokines, the HPA axis, and monoamine neurotransmitters caused by CUMS, suggesting its significant regulatory effect on this network and thereby exerting antidepressant activity.

Stress induced hippocampal apoptosis has been widely observed in depression (Lucassen et al., 2006). CUMS increased neuronal apoptosis in hippocampus and cerebral cortex of adult rats which might be associated with reduced AKT and increased ERK signaling (Parul et al., 2021). Furthermore, pro-inflammation cytokines disrupted neuroplasticity and also involved in depression behavior (Tang et al., 2016). Pro-inflammatory cytokines could activate microglia, and the activated microglia then increase apoptosis of neuronal cells (Chesnokova et al., 2016). Pro-inflammatory cytokines such as IL-1β may also induce neuronal apoptosis directly (Fan et al., 2018). The pro-apoptotic protein BAX induces programmed cell death by permeabilizing the outer mitochondrial membrane, which triggers the initiation of the caspase cascade, while Bcl-2 can form heterodimers with Bax and thereby suppressing the occurrence of apoptosis (Spitz and Gavathiotis, 2022). It was reported that icariin could prevent depression-like behaviors in CUMS-induced rats by inhibiting the Bax/cytoplasmic C/caspase-3 pathway and alleviating neuronal apoptosis (Wu et al., 2023). Similarly, JNW could upregulate Bcl-2 expression and downregulate Bax expression at both the mRNA and protein levels, which suggests that it may further inhibit hippocampal apoptosis and improve depressive symptoms, with pro-inflammatory cytokines potentially playing a role in this process.

NLRP3 plays a crucial role in mediating the inflammatory response within the central nervous system. The activation of the NLRP3 inflammasome in microglia is pivotal during neuroinflammation, as it leads to the synthesis and release of IL-1β and IL-18, thereby contributing to an elevation in inflammatory cytokines (Herman and Pasinetti, 2018). Furthermore, genetic knockout of GSDMD, Caspase-1, and the NLRP3 inflammasome in mice has been shown to ameliorate depressive-like behaviors (Li et al., 2021). Endogenous full-length NLRP3 remains in a resting state by forming a double-ring cage that is primarily localized to the membrane. Upon stimulation, NLRP3 recruits ASC via homotypic pyrin domain (PYD)-PYD interactions. Subsequently, ASC recruits Caspase-1 through caspase recruitment domain (CARD)-CARD interactions, facilitating the self-cleavage of Caspase-1 and activating the downstream signaling cascade. For example, this process results in the conversion of inactive precursors, pro-IL-1β and pro-IL-18, into their active forms, IL-1β and IL-18 (Xia et al., 2023).

While in a resting state, NLRP3 is believed to exist in concentrations insufficient to trigger inflammasome activation, and NF-κB plays a role in priming the NLRP3 inflammasome (Roy et al., 2023). Activation of Toll-like receptor, TNF receptor or IL-1 receptor initiates NF-κB signaling pathway, which results in the phosphorylation and degradation of IκBα. Following this process, NF-κB is enabled to translocate into the nucleus, where it promotes the transcription of target genes such as NLRP3 and pro-IL-1β (Yu et al., 2020). It was found that JNW notably suppressed NLRP3 and Caspase-1 mRNA expression. Western blot analysis revealed that JNW markedly enhanced the phosphorylation and degradation of IκBα and suppressed the NF-κB p65 expression, while concurrently reducing the protein expression of NLRP3 and Caspase-1. Based on these findings, it is evident that JNW could suppress the activation of the NF-κB/NLRP3 pathway induced by CUMS, which may represent a crucial mechanism underlying its antidepressant effects.

Previous studies have reported that some constituent herbs of JNW exhibit antidepressant effects. For example, *Angelicae sinensis radix* could regulate the lipid metabolism alteration induced by CUMS though sphingolipid metabolic pathways and alleviate neuroendocrine-immune network disorder (Gong et al., 2024). Meanwhile, *Angelicae sinensis radix* extracts exerted antidepressant effects by upregulating the expression of the BDNF protein and the phosphorylation of ERK 1/2 and CREB (Shen et al., 2016). *Cistanches herba* extract restored brain level of 5-HT and BDNF expression, and modulated the relative abundance of gut microbiota and the concentrations of acetate and hexanoic acid in CUMS rats (Li et al., 2018). Administration of *Schisandrae chinensis fructus* showed a promising therapeutic effect on depression induced by CUMS though activation of BDNF and upregulation of TrkB/CREB/ERK and PI3K/AKT/GSK3β/mTOR signaling pathways (Yan et al., 2019). Salvianolic acid B, the metabolite of *Salviae miltiorrhizae*, abolished chronic mild stress-induced depression through suppressing oxidative stress and neuro-inflammation via regulating NLRP3 inflammasome activation (Huang et al., 2019). These studies suggest that as a compound traditional Chinese medicine composed of 19 medicinal materials, JNW’s antidepressant effects may involve not only the additive effects of individual herbs but also potential synergistic interactions among them. Other antidepressant mechanisms reported in different traditional Chinese medicines, including modulation of the gut-brain axis function, improvement of neuronal damage, and promotion of neurotrophic factor production, may also be present in JNW and warrant further investigation. However, in this study, the mechanism by which JNW alleviates depressive symptoms, through inhibition of the NF-κB/NLRP3 pathway, suppression of inflammatory cytokine production, and improvement of HPA axis and monoamine neurotransmitter system function, holds significant value and provides an important foundation for future research.

## 5 Conclusion

In summary, the current study highlighted the antidepressant effect of JNW, demonstrating its ability to alleviate depression-like behaviors in CUMS-induced mice. Additionally, JNW could suppress pro-inflammation cytokines expression, and also restore the dysfunction of HPA axis, elevate the level of monoamine neurotransmitters level both in serum and hippocampus, inhibit hippocampal apoptosis. Furthermore, the present investigation revealed that these effects may be mediate through NF-κB/NLRP3 pathway. Thus, the current research confirmed the JNW’s antidepressant effects and elucidated the possible mechanisms. Nevertheless, further investigation is still necessary to identify the effective compounds within JNW and to fully understand its complex regulatory mechanisms for optimal clinical utilization.

## Data Availability

The original contributions presented in the study are included in the article/supplementary material, further inquiries can be directed to the corresponding author.

## Abbreviations

5-HT, serotonin; ASC, apoptosis associated speckle-like protein; ACTH, adrenocorticotropic hormone; Bcl2, B cell lymphoma/leukelia-2; Bax, Bcl-2 Associated X; CARD, caspase recruit domain; CRH, corticotropin releasing hormone; CSA, coat state assessment; CORT, Corticosterone; CUMS, chronic unpredictable mild stress; FST, forced swimming test; GSDMD, gasdermin D; HPA, Hypothalamic-pituitary-adrenal; IL, interleukin; IκB, inhibitor of NF-κB; JNW, Jiannao Pills; NE, norepinephrine; NF-κB, nuclear factor kappa B; NLRP3, nucleotide-binding oligomerization domain (NOD)-like receptor protein 3; OFT, open field test; PYD, pyrin domain; qRT-PCR, Quantitative Reverse Transcription Polymerase Chain Reaction; SPT, sucrose preference test; TCM, traditional Chinese medicine; TNF, tumor necrosis factor; TST, tail suspension test.
